# Diet change affects intestinal microbiota restoration and improves vertical sleeve gastrectomy outcome in diet-induced obese rats

**DOI:** 10.1007/s00394-020-02190-8

**Published:** 2020-02-14

**Authors:** Joana Rossell, Björn Brindefalk, Juan Antonio Baena-Fustegueras, Julia Peinado-Onsurbe, Klas I. Udekwu

**Affiliations:** 1 Department of Biochemistry and Biomedicine, Biology Faculty, Barcelona University, Barcelona, Spain; 2 Department of Molecular Biosciences, The Wenner-Gren Institute, Stockholm University, Stockholm, Sweden; 3 Surgery Unit, Arnau de Vilanova Universitary Hospital, Lleida University, Lleida, Spain

**Keywords:** Bariatric surgery, High-fat diet, Rat models, Microbiota

## Abstract

**Purpose:**

Obesity, a worldwide health problem, is linked to an abnormal gut microbiota and is currently most effectively treated by bariatric surgery. Our aim was to characterize the microbiota of high-fat fed Sprague–Dawley rats when subjected to bariatric surgery (i.e., vertical sleeve gastrectomy) and posterior refeeding with either a high-fat or control diet. We hypothesized that bariatric surgery followed by the control diet was more effective in reverting the microbiota modifications caused by the high-fat diet when compared to either of the two factors alone.

**Methods:**

Using next-generation sequencing of ribosomal RNA amplicons, we analyzed and compared the composition of the cecal microbiota after vertical sleeve gastrectomy with control groups representing non-operated rats, control fed, high-fat fed, and post-operative diet-switched animals. Rats were fed either a high-fat or control low-fat diet and were separated into three comparison groups after eight weeks comprising no surgery, sham surgery, and vertical sleeve gastrectomy. Half of the rats were then moved from the HFD to the control diet. Using next-generation sequencing of ribosomal RNA amplicons, we analyzed the composition of the cecal microbiota of rats allocated to the vertical sleeve gastrectomy group and compared it to that of the non-surgical, control fed, high-fat fed, and post-operative diet-switched groups. Additionally, we correlated different biological parameters with the genera exhibiting the highest variation in abundance between the groups.

**Results:**

The high-fat diet was the strongest driver of altered taxonomic composition, relative microbial abundance, and diversity in the cecum. These effects were partially reversed in the diet-switched cohort, especially when combined with sleeve gastrectomy, resulting in increased diversity and shifting relative abundances. Several highly-affected genera were correlated with obesity-related parameters.

**Conclusions:**

The dysbiotic state caused by high-fat diet was improved by the change to the lower fat, higher fiber control diet. Bariatric surgery contributed significantly and additively to the diet in restoring microbiome diversity and complexity. These results highlight the importance of dietary intervention following bariatric surgery for improved restoration of cecal diversity, as neither surgery nor change of diet alone had the same effects as when combined.

**Electronic supplementary material:**

The online version of this article (10.1007/s00394-020-02190-8) contains supplementary material, which is available to authorized users.

## Introduction

The gut microbiota is considered a metabolic organ consisting of more than 500 species of bacteria, viruses, and other organisms living in our intestines, involved in the host intestinal immunity and integrity [1]. Additionally, the gut microbiota has metabolic functions, regulating homeostasis and modifying the capacity for energy harvesting and has thus been proposed as a contributor to the development of obesity [2–4].

Along with producing dietary-induced obesity (D) in rodents [5], the high-fat diet (HFD) causes alterations in the microbial community assemblage when compared to control animals [6–8]. Obese animals have lower microbial diversity and perturbed abundances of the major gut phyla, *Firmicutes* and *Bacteroidetes*. It has been demonstrated that these changes in diversity are more dependent on diet than on weight gain or obesity itself [7, 9, 10] and can be reversed with calorie/fat restricted diets [11, 12]. The ratio of *Bacteroidetes:Firmicutes* was previously correlated to obesity [2, 6] but new analyses claims that no exact relationship can be established [13, 14]. Nevertheless, these alterations lead to a dysbiotic state, resulting in leaky gut and metabolic endotoxemia (i.e., low grade elevation of plasma LPS), potential drivers of the inflammatory response characteristic of obesity [9, 15, 16].

Bariatric surgery (BS), mainly Roux-en-Y gastric bypass (RYGB) and vertical sleeve gastrectomy (VSG), is presently the most effective treatment for obesity. Both procedures have similarly successful results despite being anatomically different: VSG consists of stomach resection and unchanged intestinal tract, and RYGB consists of stomach resection and modified intestinal tract, the first part of the small intestine being bypassed causing also malabsorption [17-21]. The main benefits associated with BS are a significant and sustained weight loss and improved insulin resistance [22]. However, BS is also associated with several potential complications depending on the specific surgery: RYGB is associated with increased risk of malnutrition and blood glucose fluctuations, as well as being a more complicated surgery; VSG patients have higher risk of developing gastroesophageal reflux disease [23–25]. Due to the similar benefits with less severe complications VSG popularity as the preferred BS is increasing [26]. Studies in both animal models and humans have shown that BS causes changes in the microbial community, several of which show apparent correlation with the health improvements seen following BS [8, 27–30].

This is the continuation of a previous article, where rats after being fed either a control diet or HFD for 8 weeks underwent either no surgery, simulated (Sham) surgery, or VSG [18]. Half of the HFD-fed rats were then changed to the control diet for the remaining 4 weeks, emulating dietary recommendations for weight loss (increased fruit and vegetables, reduced fat) after BS [31, 32]. We previously found that the combination of diet change and VSG in rats exerted a major effect on the weight of body and organs, reducing them to control levels. Due to the relationship between obesity and gut microbiota previously described, we decided to analyze the effects that diet and surgery had on the gut microbiome itself. The aim of the current study was to analyze the cecal microbiota using 16S RNA analysis, and describe what effect the experimental parameters—HFD, dietary switch, surgery, or combinations of the above—had on the gut microbiome.

## Materials and methods

### Animals

The animal protocol was approved by the Ethical Committee for Animal Experimentation of the University of Lleida (CEEA. 04–05/12). Male Sprague–Dawley rats (9 weeks old, weight 315.7 ± 5.4 g) from the breeding house of the University of Lleida were pair-housed in polypropylene cages under controlled conditions (22 °C, 12/12-h day-night cycle, 40–78% humidity).

### Study design

**Fig. 1:**
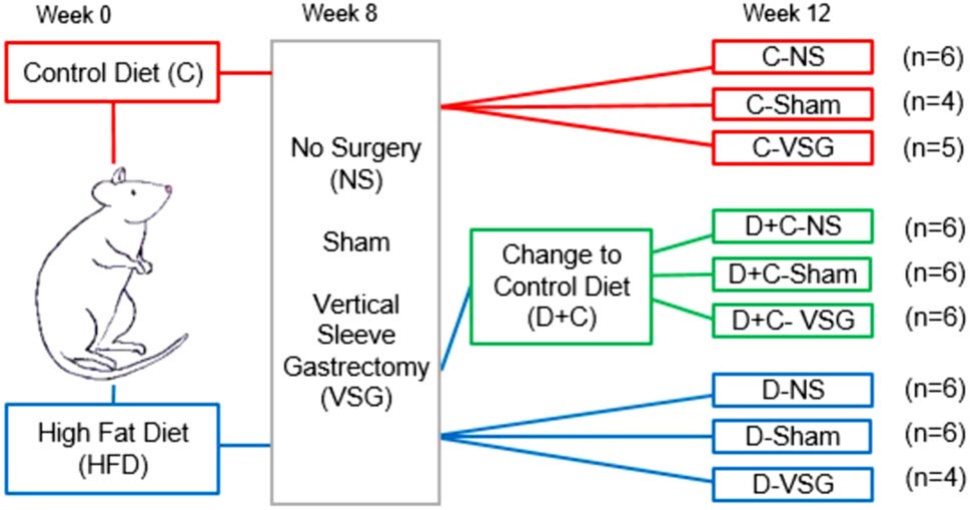
Experiment design and group distribution. Rats were fed either the control diet (red line) or HFD (blue line) for 8 weeks. At week eight, each diet group was then divided in three (*n* = 6), and subjected to one of the three surgical situations: No Surgery, Sham surgery or VSG. Half of the HFD-fed rats were then switched to the control diet (green line). Rats continued the allocated diet until week 12

Eighteen animals were fed a control diet (C group) with a calorie composition of 20% protein, 67% carbohydrate, and 13% fat (Tekland Global, 2014C, Envigo), and 36 animals were fed a HFD (D and posterior D + C group) with a calorie composition of 18% protein, 21% carbohydrate, and 60% fat (TD.06414, adjusted calories 60/fat, Envigo). Detailed composition of both diets is found in Online Resources 1. Food and water were given ad libitum. After 8 weeks, animals were divided into three groups (six C and 12 D animals per group) and underwent no surgery (NS), Sham, or VSG. Animals continued on their designated diet for four more weeks, apart from six animals of each D group that were then switched back to control diet (D + C), establishing the following subgroups: C-NS, C-Sham, C-VSG, D-NS, D-Sham, D-VSG, D + C-NS, D + C-Sham, and D + C-VSG (Fig. 1). VSG and sham interventions were performed according to previously described procedures [18]. In brief, VSG animals had 70–80% of their stomach removed under anesthesia while Sham animals underwent the same operative procedure but their stomach remained intact. Both Sham and VSG animals received antibiotic treatment (Enrofloxacina, 10 mg/kg every 12 h) for 5 days (2 days pre-surgery as a prophylactic treatment, and the following three days post-surgery). Surgery had a mortality rate of 9.25% during the first two days post-surgery.

### Sample collection

Animals were sacrificed by decapitation at week 12 after a 12 h fast. Blood samples were collected in tubes containing EDTA and plasma was obtained through centrifugation (2500 rpm, 15 min, 4 °C). Caeca were collected, snap-frozen in liquid nitrogen and stored at − 20 °C. Epididymal and perirenal adipose tissues were collected, weighed and stored at − 20 °C.

### DNA extraction

DNA was extracted from cecum samples using either the QIAamp Fast DNA Stool Mini kit or the DNA easy Power Soil Kit (QIAGEN, Hilden, Germany). The concentration of DNA was assessed using the Quant-iT PicoGreen dsDNA Assay kit (ThermoFisher, Massachusetts, USA). DNA samples were stored at − 20℃.

### Sequencing and analysis of 16S amplicon data

Isolated DNA was amplified with forward primer 341F: (CCTACGGGNGGCWGCAG) and reverse primer 805R: (GGACTACHVGGGTWTCTAAT) targeting the V3—V4 hypervariable region of the coding sequence for the 16S small ribosomal RNA, rRNA. Amplified DNA was sequenced on an Illumina MiSeq machine, using the MiSeq v3.0 reagent kit leading to 2 × 300 bp paired-end reads. Initial demultiplexing was done with the default Illumina MiSeq Control Software (2.6.2.1). Down-stream quality control, trimming, filtering, merging of forward and reverse reads, chimera removal and identification of amplicon sequence variants (ASV) was performed with the R software version 3.4.2 (https://www.R-project.org), using the DADA2 R package version 1.6 [33, 34]. Metabolic reconstruction from 16 s amplicon data of KEGG pathways ko04973 (carbohydrate digestion and absorption) and ko00071 (fatty acid degradation) was done from normalized to even depth ASV abundance tables for the top 100 most abundant taxa created using the above described method, followed by submission to the Piphillin server using the KEGG database, release of October 2018, and a cut-off sequence similarity value of 95% [35].

### Biochemical parameters

Glucose was enzymatically analyzed in a METROLAB 2300 auto-analyzer (RAL, Laboratory Techniques, Spain); glucagon and leptin were measured using an ELISA kit (R&D, USA), insulin and unacylated ghrelin were measured using an ELISA kit (Bertin Bioreagent, France), all according to the manufacturer’s protocols.

### Glucose homeostasis indicators

Glucose homeostasis was measured by the insulin sensitivity index (ISI), the homeostatic model assessment of insulin resistance (HOMA-IR) and the homeostatic model assessment of β cell function (HOMA-β) calculated according the formulas: ISI = 1/fasting glucose fasting insulin; HOMA-IR = fasting insulin fasting glucose/22.5; HOMA-β = 20 $\times$ fasting insulin/(fasting glucose − 3.5).

### Data analysis and statistics

Body weight gain (BWG) was calculated by subtracting initial body weight from measured body weight at posterior time. The adiposity index was calculated as the sum of epididymal and perirenal adipose tissues/body weight $\times$ 100. Body weight values were expressed as mean ± SEM. Differences in BWG were analyzed by a repeated measure ANOVA with Mauchly’s sphericity test followed by GG corrections, using the R software. Body weight gain at week 12 and biochemical parameter differences were analyzed by a two-way ANOVA followed by a Tukey post-test. Graphics and statistical analysis of gut microbiota were done with the Phyloseq package version 1.20.0 [36]. Taxa were expressed as relative abundances with expressed values as the mean of each group. The Alpha diversity was determined using Shannon and Simpson indices and differences were analyzed by a two-way ANOVA followed by a Tukey post-test. Beta diversity was estimated by Non-Metric Multidimensional Scaling (NMDS) and differences were analyzed by PERMANOVA. The DeSeq2 R package [37] was used to analyze differentially abundant taxa on genus level (false discovery rate (FDR) < 0.01 and log_2_-fold change (log_2_ FC) >|10|) associated with the various combinations of diet and surgery. The correlation analysis between differentially abundant genera and biochemical parameters was assessed by Spearman’s correlation method using the R software, coefficients were plotted on a heatmap using the corrplot package. Differences were considered significant when *P* values < 0.05.

## Results

### General parameters

**Fig. 2:**
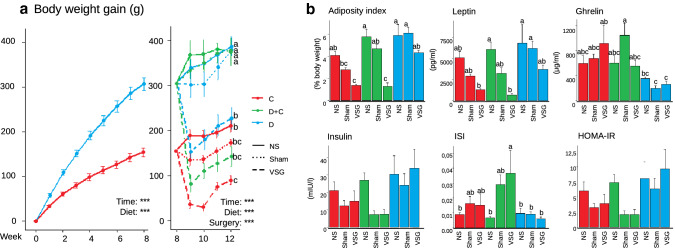
**a** The D group had a higher body weight gain from week one (*P* < 0.001). At week eight, half of the D animals switched from the HFD to the control diet, and all were divided into surgery groups NS, Sham or VSG. At week 12, VSG had a significantly lower BWG than Sham or NS in the same diet group, especially for D + C (*P* < 0.001). **b** Different parameters at week 12. The adiposity index was lowered by the combination of VSG and diet switch. Leptin was affected by VSG. Ghrelin was reduced in D groups. Insulin Sensitivity Index increased in D + C-VSG. No significant changes were seen in Insulin or HOMA-IR. *P* values < 0.001 (***) and **a**–**c** correspond for significantly different groups (Tukey post-test)

Results on body and organ weight were previously published [18] and are thus not described in detail here. Body weight gain was significantly higher after 1 week of HFD in the D group (*P* < 0.001) and continued to be so during the whole experiment (except D-VSG and D + C-VSG). At the end of the experiment, the BWG for D-VSG was close to C-NS, while D + C-VSG was similar to both C-Sham and C-NS (Fig. 2a). Adiposity index was similar for all the NS groups, D + C-Sham, D-Sham, and D-VSG, higher than in C-VSG and D + C-VSG. Leptin decreased significantly in C-VSG and D + C-VSG groups, while Ghrelin was lower in all the D groups. The maintained HFD tended to increase insulin levels and thus HOMA-IR but was not significant. Surgery had some effect only for the C and the D + C groups, especially with respect to ISI, were D + C-VSG showed the best insulin sensitivity (Fig. 2b).

### Diversity

**Fig. 3:**
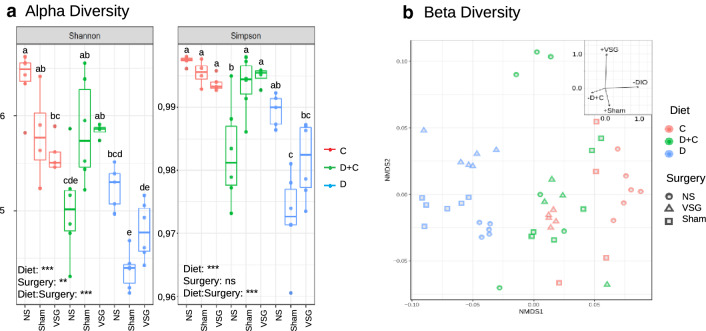
Diversity measures. **a** Shannon and Simpson indices showing sample alpha diversity. The bottom and top of the boxplot indicate the first and third quartile, whilst the line inside the box show the median. Diversity was reduced by both Sham surgery and VSG in all groups. D samples had the lowest diversity except for D + C-NS in the NS situation. *P* values < 0.001 (***), *P* values < 0.01 (**) and **a**–**c** correspond for significantly different groups (Tukey post-test). **b** Beta diversity. The non-metric Multidimensinal Scaling (NMDS) plot for the bacterial communities in our samples based on Bray–Curtis dissimilarities. D groups, C-NS and C-VSG formed distinct clusters. PERMANOVA analysis: Surgery (*P* < 0.001), Diet (*P* < 0.001). D + C samples formed less defined clusters, but were very distinct from D samples and were overlapping the C-VSG and C-Sham groups

Alpha diversity—diversity in each group, calculated by Shannon and Simpson Indices—ranked the D groups as the least diverse, and the control C-NS as the most diverse (Fig. 3a). Sham and VSG surgery negatively affected diversity in C and D-groups, but combined with the dietary switch, increased diversity for D + C. Taken as a whole, diet was the main factor affecting alpha diversity, together with the combination of diet and surgery, while surgery alone had less of an effect (and no effect in the Simpson Index) (Two-way ANOVA, *P* < 0.01).

Non-Metric Multidimensional Scaling (NMDS), based on Bray–Curtis dissimilarities, was used to assess the beta-diversity—differences in taxonomic abundances between samples—(Fig. 3b) showing that diet and VSG had a very significant impact (*P* < 0.001, PERMANOVA). Constrained ordination showed diet as the strongest factor separating samples on the first, most explanatory axis, and thus, the strongest factor driving the separation of populations between C and D groups. The D samples formed a distinct, and more defined, group compared to the other two diets. Interestingly, the D + C groups showed less defined clustering, with samples scattered intermediately between clusters representing the D and C cohorts, most evident for D + C-Sham and D + C-VSG, which overlapped with the C-VSG group. Surgery also had a significant impact, resulting in a tight clustering for C-VSG when compared to the respective NS and Sham.

### Modifications in relative abundances of cecal microbes

**Fig. 4:**
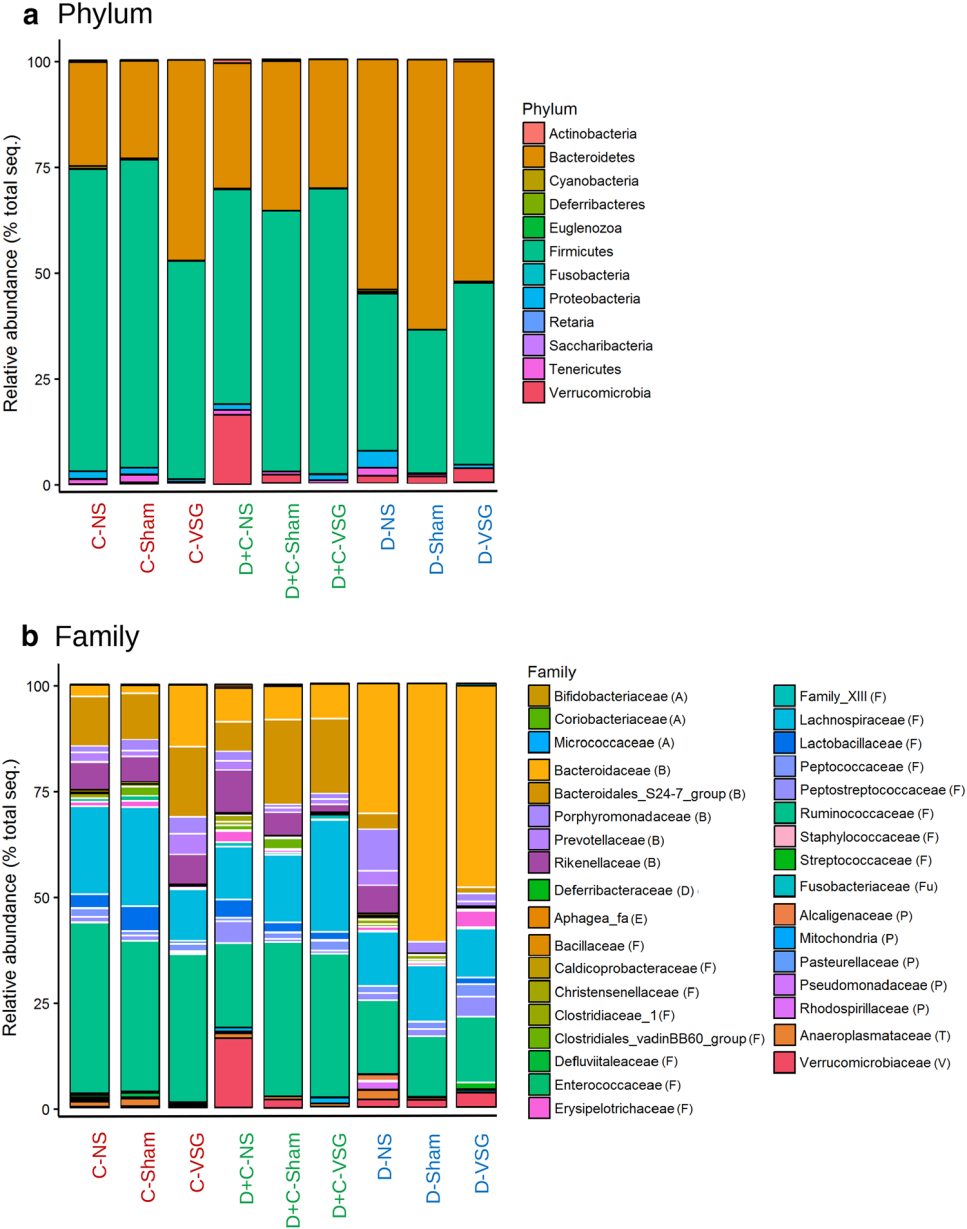
Relative abundances of bacterial composition, **a** at the phylum level, dominated by the *Bacteroidetes* and the *Firmicutes* phyla. Relative abundances of bacterial composition, **b** at the family level, with phyla separations marked with black lines and family separations marked with white lines. Family names in legend grouped by phyla (phylums initial letter). Group labels were marked according the diet: Red for C, Blue for D, Green for D + C

Diet and surgery induced substantial differences between groups. The microbiota was dominated by the phyla *Bacteroidetes* and *Firmicutes*, which accounted for more than 80% of the microbiome in all groups (Fig. 4a). On average, the maintained HFD in D increased the *Bacteroidetes* fraction, but this fraction was normalized after the switch to the control diet in D + C groups. The diet change also increased the relative abundance of *Verrucomicrobia*, up to 16.31%, in the D + C-NS, but not in the antibiotic treated D + C-Sham and D + C-VSG groups. Importantly, these two groups showed a strong similarity at the phyla level to the C-NS and C-Sham groups. On the other hand, surgery increased the relative abundance of *Firmicutes* in D-VSG compared with D-NS (42.43% vs 37.10%, resp.), while reducing *Proteobacteria* (0.87% vs 4.05%, resp.). In the C groups, no substantial differences were seen between C-NS and C-Sham, but VSG reduced the *Firmicutes* levels (51.41%). At the family level (Fig. 4b), the D groups had higher *Bacteroidaceae* abundance, drastic reductions in *Bacteroidales_S24-7* (less than 5%) and reduced *Ruminococcaceae* abundance (also observed in D + C-NS) compared with C. The family *Rikenellaceae* was reduced after Sham or VSG, but only in HFD-fed groups. D + C-NS and D-VSG similarly had higher levels of *Erysipelotrichaceae* (2.65% and 3.92%). VSG reduced the amount of several families in the *Firmicutes* phylum, such as *Christensenellaceae*, *Clostridiaceae*, *Clostridiales*, and *Defluviitalaceae*. Diet and surgery significantly increased or decreased some genera (mainly belonging to the *Firmicutes* phylum) when compared to their respective C group (Online Resource 2). Several *Ruminococcaceae* decreased in both D and D + C in the NS groups, as well as several genera belonging to *Lachnospiraceae* (*Acetifactor*, *Cellulosilyticum*, and *Lachnospiraceae_NK4A136_group*) which also increased in D + C-Sham. The groups D + C-VSG, D-Sham and D-VSG had fewer significantly different genera with respect to their control matching groups. The *Lachnospiraceae_NK4A136_group* was common in all groups but responded differently to each experimental situation.

### Correlation between cecal bacteria and other parameters

**Fig. 5:**
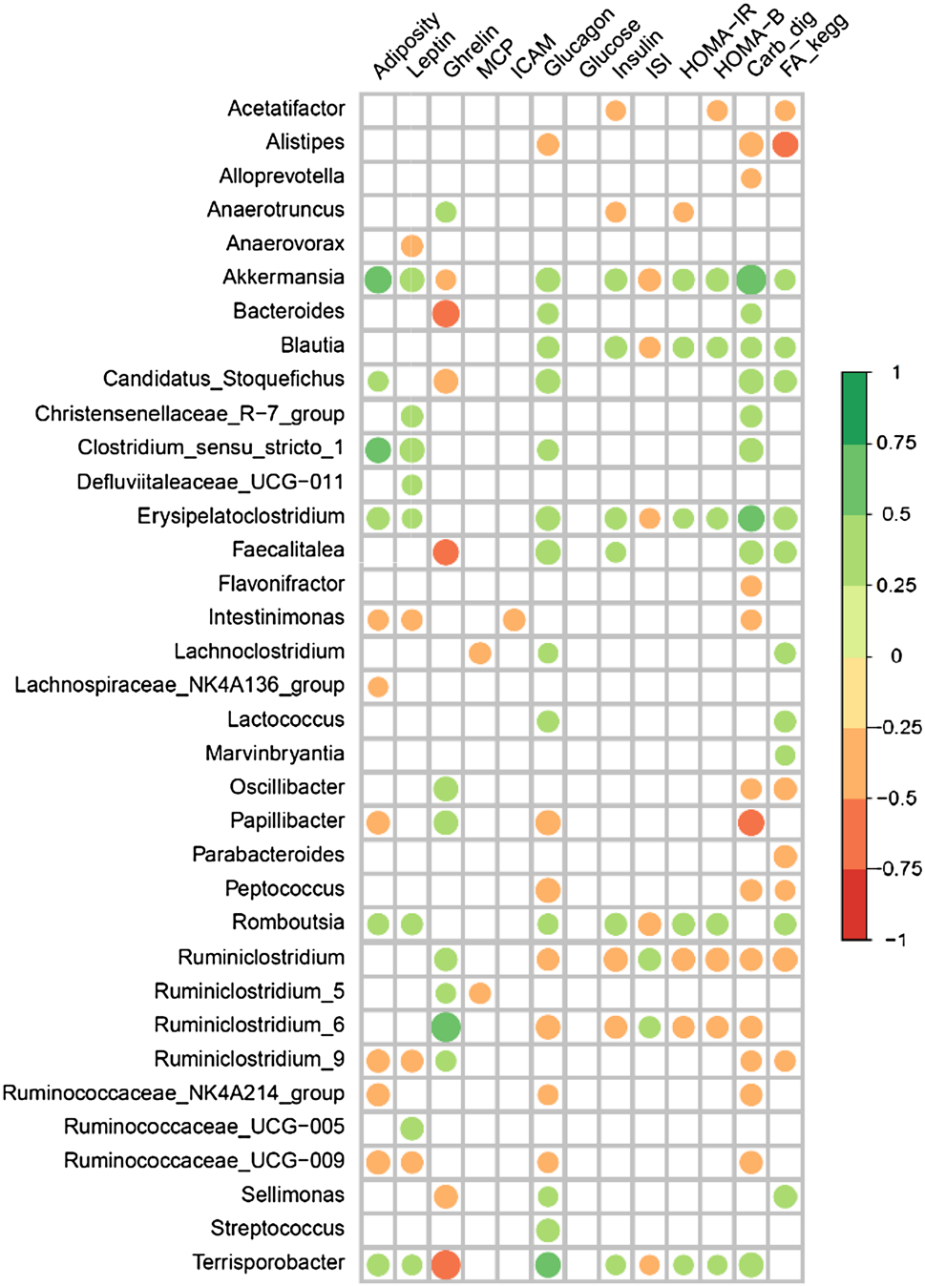
Graphic representation of the Spearman correlation coefficients between the significantly altered genera obtained by DESeq and different parameters such as adiposity index, biochemical parameters, and KEGG pathways. Positive correlations are shown in green color and negative correlations in red color. The color intensity and the circle size are proportional to the correlation coefficients. Only genera with significant correlations (< 0.05) are shown

To observe possible associations between the 30 genera showing highest change in the dataset showing significant change and diverse biological parameters, we performed a correlation analysis between them. Figure 5 shows a plot of Spearman’s correlation coefficient for significant correlations. We observe that the genus *Akkermansia* strongly correlated to the adiposity index and to the carbohydrate digestion pathway related to the formation of short chain fatty acids. *Erysipelatoclostridium* had similar correlations in addition to *Blautia*. *Bacteroides*, *Faecalitalea*, and *Terrisporobacter* being inversely correlated with ghrelin.

## Discussion

In this work we investigated the effect of HFD, VSG (accompanied by dietary switch or not) and change of diet alone on the Sprague–Dawley rat cecal microbiota, a follow-up of our earlier work reporting on weight loss results [18]. The combined effect of diet and surgery had distinctive effects on the microbiota in line with previous reports [8, 28, 30]. In addition to this, an obvious effect attributable to pre-operative antibiotic prophylaxis is noted, reflected in the effects observed in the Sham-operated cohort of this study. This reasoning is entirely in line with the ecological theory where, at the species level, functional redundancy is prevalent in such complex communities. However, the significance is downplayed by therapy-oriented efforts in attributing pathophysiology to individual microbes, such as in the absence of a direct effect (e.g., toxin production by the causative pathogen) where the community function (host phenotype) may not be reliant on a singular organism. We have thus exclusively approached how surgery (VSG, Sham surgery or, No Surgery) combined with dietary variables (control diet, HFD, or HFD switched to control diet post-operatively) affect the gut microbiota.

The relative abundance of the main phyla was strongly affected by diet, and high levels of *Bacteroidetes* accompanied by concomitant low levels of *Firmicutes* were noted for the D cohort (Fig. 4a) as shown in previous studies [10, 38]. The maintained HFD also altered diversity, separating the D populations from both C and D + C, no matter the surgical approach (Fig. 3b), and lowered the alpha diversity (Fig. 3a), an indication of dysbiosis [39]. The HFD is said to have a lesser negative influence on the alpha diversity than other obesogenic diets (such as the one mimicking the western diet used by Bortolin et al. [40]. Nevertheless, we observed a pronounced reduction that persisted after the switch of diet alone. Alpha diversity was also affected by VSG, but to a lesser degree than by the change of diet, although VSG only removes the glandular part of the stomach and leaves the intestine intact [28]. Nonetheless, both C and D groups subjected to VSG had reduced alpha diversity, similar to what has been seen in humans and animals [41, 42], but also to Sham surgery, suggesting the effects of the antibiotic Enrofloxacina were the primary cause rather than the surgery itself. Interestingly, the combination of antibiotic and diet change increased alpha diversity for D + C-Sham and D + C-VSG. This could imply that a wipeout effect from the antibiotic was needed to achieve a more beneficial microbiota restoration after the change of diet. This highlights the strong, but often-neglected effect of pre-operative antibiotics on the microbiome (highlighted in a recent review [43]). More research is thus warranted towards further understanding of how antibiotic choices may be amenable pre-operatively in the re-establishment of a healthy microbiota.

The D + C groups were of particular interest in this study as the diet switch to the control diet, even in the absence of other factors, resulted in major compositional differences in the HFD-fed rats showing a partial restoration of the original microbiota. This is in line with other studies [4] where a significant reduction in the relative abundance of *Bacteroidetes* and an increase in *Firmicutes* compared to D was reported. As we showed previously [18], ‘recovery’ (i.e., net weight and adipose tissue reduction) was better achieved in the group combining diet switch and VSG. In this study, we observed improvements in BWG, adiposity index, leptin, ghrelin and ISI, returning to control values in the D + C-VSG group (Fig. 2a, b). Furthermore, D + C-VSG rat microbiomes had a closer resemblance to the C groups on the phylum level (Fig. 4a) but still differed at family level (Fig. 4b), proving the difficulties of proper restoration of the microbiota after a diet-induced perturbation, especially at lower taxonomy levels, as seen in other studies with HFD-fed rodents [6, 15, 44]. Once more, this remains a strong indicator of community-level synergistic effects, and argues against single microbial species causality.

While not disregarding the possibility of redundant species function, changes in abundance of specific taxa warrants attention; *Akkermansia muciniphila*, the only member of *Verrucomicrobiaceae*, is described as a marker of improved host health [45]. This species was elevated in D + C-NS (Fig. 4b) and seems to be directly affected by antibiotic administration, as levels were not increased in D + C-Sham or D + C-VSG. *A. mucinphila* was positively associated with several parameters related to obesity (Fig. 5) but also with increased adiposity. Several of the genera found in Fig. 5 contain species that produce short chain fatty acid (SCFA). The SCFAs are involved in the maintenance of the intestinal epithelium and have been associated with obesity and its comorbidities, alongside with improvements in intestinal inflammation [46–50]. Indeed, we found correlations—both negative and positive—between significantly changed genera and the chosen parameters, and we observed similar correlation patterns for the following taxa; (*Akkermansia*, *Blautia* and *Erysipelatoclostridium*, or *Ruminiclostridium* and *Ruminiclostridium-6*). Again, this may highlight the synergistic effects at the community level, instead of pin-pointing single species as causative agents.

To maximize the weight loss associated with VSG and stabilize the microbiome, a diet-switch combined with probiotic administration may maximize health gains by counteracting HFD effects and reduce body weight [39, 51]. The aforementioned *A. mucinphila* is a proposed probiotic, implicated in combating obesity and metabolic syndrome [45, 51]. Accordingly, any prospective probiotic cocktail devised to facilitate weight loss would likely require extensive testing in multiple dietary backgrounds. Another line of reasoning regarding optimal weight loss after BS is the effect of VSG on SCFA-producers, resulting in reductions of butyrate and decreased LPS translocation (leaky gut) relevant to tight junction regulation in the intestinal epithelium [40]. Clearly, more work is needed to clarify the nature of probiotics, SCFA-producers and several other organisms in host physiology, but also and perhaps more importantly, the intricate structures and relationships between and among taxa at the community level. Approaching exact numbers and true abundances will require more in-depth analyses combining deep sequencing and reverse transcriptomics to identify actively dividing populations stimulated by each intervention.

The difficulties associated with extending results from animal models to human interventions are diverse and widely acknowledged. Rodents allow for the inclusion of controls necessary for the optimal evaluation of multiple variables and their effects on gut microbiota. However, to facilitate easier comparisons in such studies, a need exists to standardize diets. Such improved diets are currently being developed in several instances [13], as the standard HFD is an inadequate representation of western food habits, believed to be responsible to some extent for the obesity pandemic. Also, an additional no-surgery antibiotic-treated group would have been useful in evaluating this effect, and opens up avenues for further investigation on future studies.

In conclusion, this study provides evidence that a controlled diet following VSG is key to increasing alpha diversity and restoring the HFD-perturbed taxonomomic composition of the microbiota. It highlights the effect of antibiotic exposure in the pre-operative stage, hinting at its importance in microbiome modulation caused by the procedure. Gut microbiota alterations may be beneficial during recovery of healthy body weight after bariatric surgery. Further studies are needed to elucidate the intricate relationship that gut microbiota, diet, weight loss, antibiotics and BS have. Modulation may represent a new plausible target in improving the outcome of interventions against obesity.

## Electronic supplementary material

Below is the link to the electronic supplementary material.

Supplementary file1 (DOCX 14 kb)

## Abbreviations

SCD: Standard chow diet
HFD: High-fat diet
D: Diet induced obesity
BS: Bariatric surgery
VSG: Vertical sleeve gastrectomy
NS: No surgery
