# Supplementary material for: Diet change affects intestinal microbiota restoration and improves vertical sleeve gastrectomy outcome in diet-induced obese rats
Source: Eur J Nutr. 2020 Feb 14;59(8):3555–64. doi: 10.1007/s00394-020-02190-8 (PMC7669806; doi:10.1007/s00394-020-02190-8)
Supplement: Supplementary file 1 — Supplementary file1 (DOCX 14 kb) [file 394_2020_2190_MOESM1_ESM.docx]

**Supplementary data**

**Supplementary Table 1. Diet composition for Control (C) and High-Fat Diet (HFD)**

|  | **C** | **HFD** |
| --- | --- | --- |
|  | **% weight** | **% weight** |
| Protein | 14.3 | 23.5 |
| Carbohydrate | 48 | 27.3 |
| Fat | 3.4 | 34.3 |
| Saturated | 0.6 | 12.48 |
| Monounsaturated | 0.7 | 16.05 |
| Polyunsaturated | 2.1 | 5.4 |

Control formula: Wheat middlings, ground wheat, ground corn, corn gluten meal, calcium carbonate, soybean oil, dicalcium phosphate, iodized salt, L-lysine, vitamin E acetate, DL-methionine, magnesium oxide, choline chloride, manganous oxide, ferrous sulfate, menadione sodium bisulfite complex (source of vitamin K activity), zinc oxide, copper sulfate, niacin, calcium pantothenate, calcium iodate, pyridoxine hydrochloride, riboflavin, thiamin mononitrate, vitamin A acetate, vitamin B12 supplement, folic acid, cobalt carbonate, biotin, vitamin D3 supplement.

HFD Formula: Casein, L-Cystine, maltodextrin, sucrose, lard, soybean oil, cellulose, mineral mix (AIN-93G-MX (94046)), calcium phosphate (dibasic), vitamin mix (AIN-93-VX 94047)), choline bitartrate, blue food color.

**Supplementary table 2. Significantly altered genera between groups**

Significant differences between groups, separated by surgery groups, at Genus level. First group Indicates a decrease compared to C, Second indicates a mixed response, and third indicates an increase compared to C. In bold, Genus present in both D+C and D.

| **C vs D+C** |  |  |
| --- | --- | --- |
| **No Surgery** | **Sham** | **VSG** |
| **Acetatifactor** Anaerotruncus Bacteroides **Cellulosilyticum** **Coprococcus**_1 Intestinimonas Mucispirillum NA Parabacteroides **Ruminoclostridium** Ruminoclostridium_5 Ruminoclostridium_9 **Ruminococcaceae_NK4A214_group** **Ruminococcaceae_UCG-009** **Ruminococcaceae_UCG-010** Ruminococcaceae_UCG-014  **Lachnospiraceae_NK4A136_group** Christensellaceae_R-7_group Ruminococcaceae_UCG-005  Akkermansia Blautia | **NA**  Akkermansia Anaerotruncus Anaerovorax **Bacteroides** Caproiciproducens Christensellaceae_R-7_group Lachnospiraceae_NK4A136_group Lachnospiraceae_UCG-6Lactococcus MarvinbryantiaRuminiclostridium Ruminiclostridium_5 Ruminiclostridium_6 Ruminococcaceae_UCG-005 Ruminococcaceae_UCG-014 Ruminococcus_1 | Lachnospiraceae_NK4A136_group |

| **C vs D** |  |  |  |
| --- | --- | --- | --- |
| **No Surgery** | **Sham** | **VSG** |  |
| **Acetatifactor Cellulosilyticum** Christensellaceae_R-7_group **Coprococcus**_1 **Ruminoclostridium Ruminococcaceae_NK4A214_group** Ruminococcaceae_UGC-005 **Ruminococcaceae_UCG-009 Ruminococcaceae_UCG-010** Ruminococcaceae_UCG-014  Anaerotruncus  Bacteroides **Lachnospiraceae_NK4A136_group**  NA Papillibacter Ruminoclostridium_9 | Alistepes Anaerostipes    Lachnospiraceae_NK4A136_group  **Bacteroides** | Alistipes Oscillobacter    Lachnospiraceae_NK4A136_group  NA  Bacteroides  Erysipelatoclostridium |  |
